# Epidemiology of Gastric Cancer—Changing Trends and Global Disparities

**DOI:** 10.3390/cancers16172948

**Published:** 2024-08-24

**Authors:** Manami Inoue

**Affiliations:** National Cancer Center Institute for Cancer Control, 5-1-1 Tsukiji, Chuo-ku, Tokyo 104-0045, Japan; mnminoue@ncc.go.jp; Tel.: +81-3-3542-2511 (ext. 3385); Fax: +81-3-3547-8578

**Keywords:** epidemiology, gastric cancer, risk factor, helicobacter pylori, survival

## Abstract

**Simple Summary:**

In overall terms, the incidence and mortality of gastric cncer have substantially decreased over the past century, attributable to decreases in risk factors such as *H. pylori* infection, tobacco smoking, and the intake of salt-preserved food. Nevertheless, obesity is ongoing and may result in an increase in cardia cancer, particularly in Western populations. Careful monitoring of this outcome is warranted in both Western and Asian populations.

**Abstract:**

Overall, the past century has seen a substantial decline in gastric cancer, attributable to decreases in risk factors such as *H. pylori* infection, tobacco smoking, and the intake of salt-preserved food. One potential preventive strategy for those at high risk is *H. pylori* eradication for infected subjects, but confirmation of this effect awaits longer follow-up. Obesity continues to advance and may cause increases in cardia cancer, particularly in Western populations, and careful monitoring of this outcome is warranted in both Western and Asian populations. These changes in gastric cancer epidemiology foreshadow a new era in gastric cancer control and warrant further monitoring of descriptive patterns and risk factors.

## 1. Introduction

The epidemiological characteristics of gastric cancer have changed dramatically in recent decades. In particular, gastric cancer is characterized by large geographical variation. This variation provides insights into how the risk factors of gastric cancer and its primary prevention should be approached. This review outlines recent global trends in gastric cancer and its risk factors and the future disease landscape over the coming decades.

## 2. Global Descriptive Epidemiological Trends

### 2.1. Current Trends

**Figure 1:**
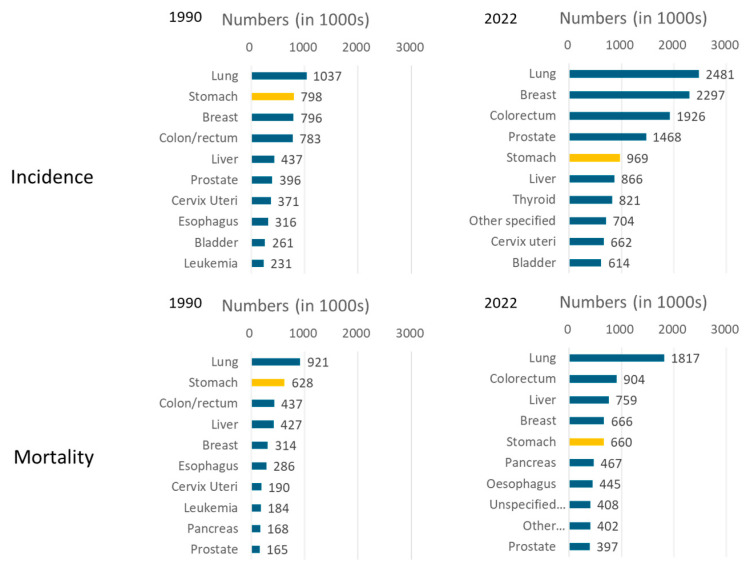
Most frequent cancers worldwide by incidence and mortality in both sexes in 1990 and 2022 [1,2].

**Figure 2:**
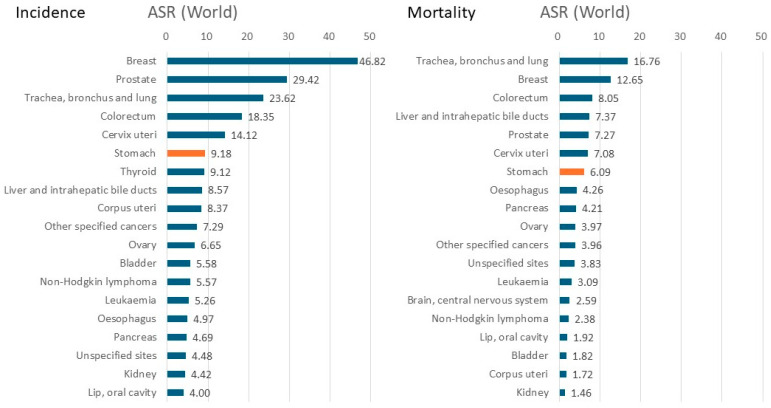
Age-standardized rate (world) of cancers per 100,000 by incidence and mortality in both sexes in 2022 [1].

According to an estimate by the International Agency for Research on Cancer (IARC) (Global Cancer Observatory: Cancer Today) [1], the number of incident cases of gastric cancer worldwide made it the fifth-most common cancer after lung, breast, colorectal, and prostate cancers in 2022. This is despite ranking second only to lung cancer in 1990 [2]. Moreover, the number of deaths in 2022 ranked fifth after lung, colorectal, liver, and breast cancers, again despite ranking second to lung cancer in 1990 (Figure 1) [1,2]. These changes make apparent a steady decline in gastric cancer, although it remains one of the major cancers worldwide. The 2022 age-standardized rate of gastric cancer worldwide ranks it fifth for incidence and seventh for mortality (Figure 2).

**Figure 3:**
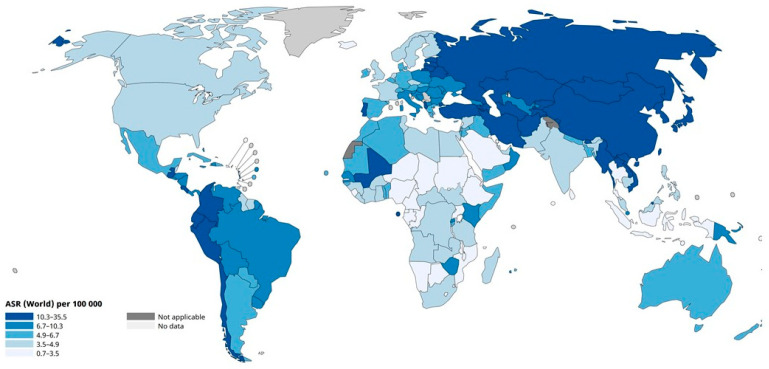
Age-standardized incidence rate (world) of gastric cancer per 100,000 in both sexes in 2022 [1]. The designations employed and the presentation of the material in this publication do not imply the expression of any opinion whatsoever on the part of the World Health Organization/International Agency for Research on Cancer concerning the legal status of any country, territory, city, or area or of its authorities or concerning the delimitation of its frontiers or boundaries. Dotted and dashed lines on maps represent approximate borderlines for which there may not yet be full agreement.

**Figure 4:**
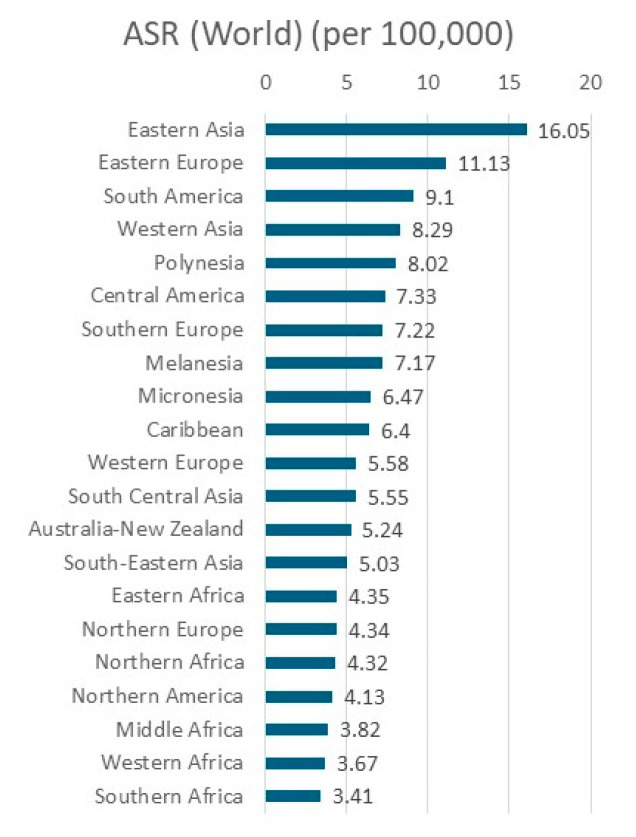
Age-standardized (world) incidence rate of gastric cancer by UN Region in both sexes in 2022 [1].

**Figure 5:**
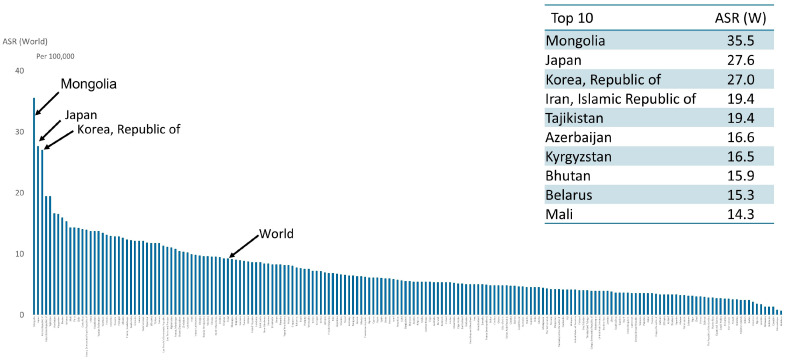
Age-standardized incidence rate (world) of gastric cancer by country ranking in both sexes in 2022 [1].

**Figure 6:**
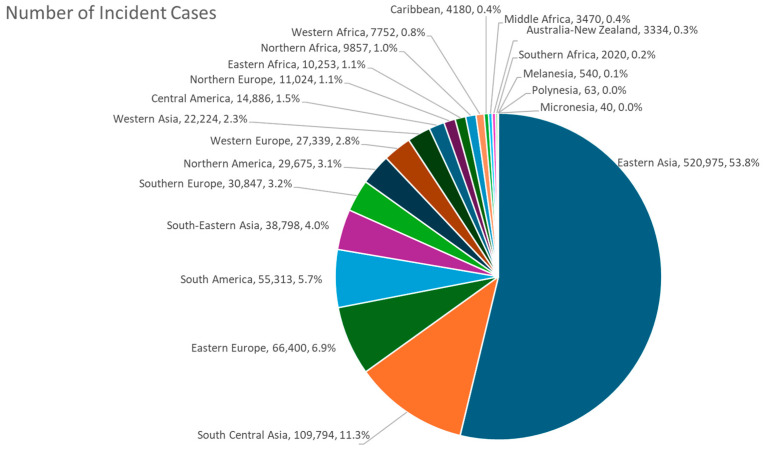
Distribution of gastric cancer by UN region by absolute numbers in both sexes in 2022 [1].

Viewed geographically, it is easily seen that gastric cancer is highly prevalent in Eastern Asia, Central and South Asia, and South America, as well as in Eastern Europe (Figure 3). On an age-standardized basis, Eastern Asia is by far the leading region, followed by Eastern Europe, South America, and Western Asia (Figure 4). Within Eastern Asia, Mongolia ranks highest in the world, followed by Japan and Korea (Figure 5). Indeed, Eastern Asia accounts for more than half of the world’s gastric cancer incident cases (Figure 6).

### 2.2. Survival Trends

**Figure 7:**
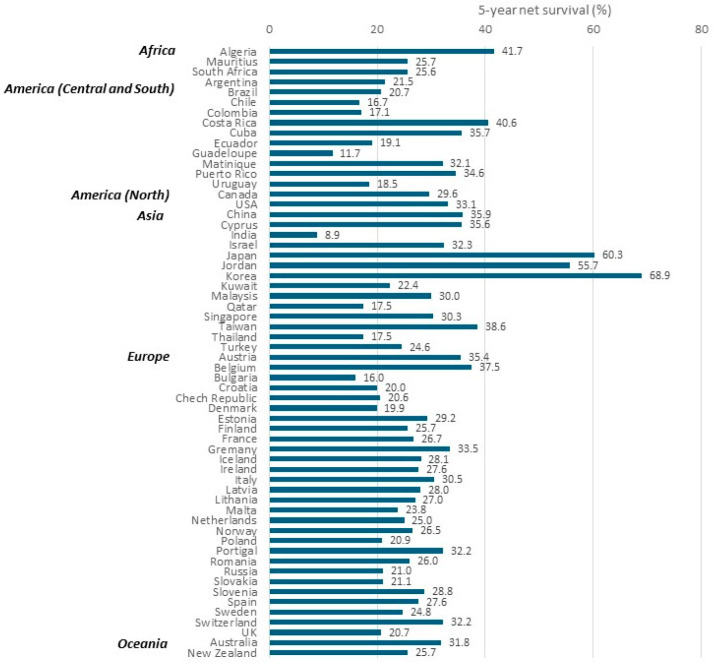
Global distribution of age-standardized 5-year net survival for adults (15–99 years old) diagnosed with gastric cancer in 2010–2014 (both sexes) [3].

The global surveillance of trends in cancer survival (CONCORD) program has long monitored global cancer survival rates and now includes more than 70 countries and 75% of all cancer cases worldwide, with high representativeness [3]. The most recent comparison, CONCORD-3, reports that the 5-year relative survival rate for gastric cancer patients diagnosed in 2000–2014 generally ranged from 20 to 40%. However, in 2010–2014—the latest period—survival was markedly high in Korea (68.9%) and Japan (60.3%) (Figure 7), showing an increase of 10% or more from 2000 to 2004. This improvement might be attributable to the organized gastric cancer screening programs conducted in both countries and the consequent early detection of gastric cancer [3].

## 3. Clinico-Epidemiological Features

### 3.1. Subsite Distribution

Gastric cancer is classified by anatomical origin into cardia and non-cardia subtypes. Some risk factors for these two types are shared, while others are subtype-specific. Regarding prevalence, the non-cardia subtype is more common in East Asian populations, where gastric cancer rates are generally high, whereas the cardia subtype has a higher prevalence in Western populations. This difference provides further clues to the difference in etiology between them.

**Figure 8:**
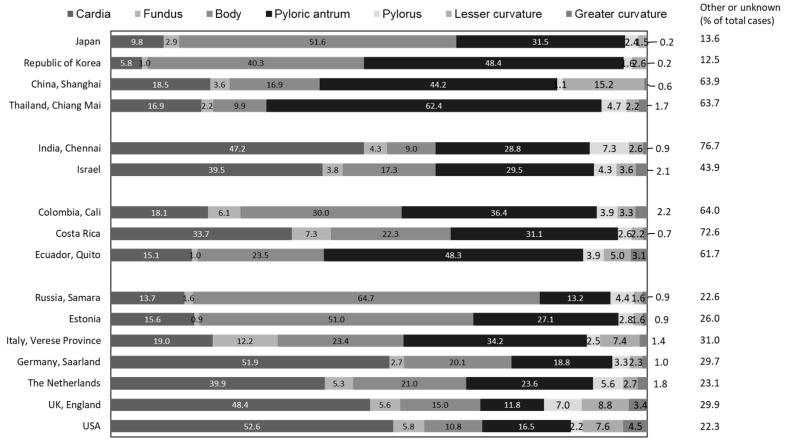
Subsite distribution of gastric cancer in males in 2013–2017 [4].

Comparing the subsite distribution of gastric cancer from the global cancer registry data [4], a higher proportion of distal gastric cancer is observed in populations such as Japan and Korea, where gastric cancer is more common, than in populations where gastric cancer is less common, including the UK and USA (Figure 8). Even among Western populations, those with high rates of gastric cancer tend to have higher rates of distal disease than other Western populations. Over the past decades, gastric cancer has markedly decreased globally, as *Helicobacter pylori* (*H. pylori*), a major cause of gastric cancer, has declined significantly. It is known that countries with a high *H. pylori* infection rate have a higher proportion of cancers in the more distal part of the stomach than countries with a low *H. pylori* infection rate.

### 3.2. Epidemiological Trends in Esophagogastric Junction Cancer

Adenocarcinoma of the esophagogastric junction has recently increased in Western countries. A review of annual trends by detailed site using the US SEER program data showed a particular increase in cancer at the esophagogastric junction, from 1.22 cases per 100,000 persons around 1975 to 2.05 in the early 1990s, followed by a leveling off [5]. The reasons may include a decrease in *H. pylori* infection as well as an increase in obesity and associated esophagogastric reflux disease.

More fundamentally, however, cancers occurring at the esophagogastric junction have been ambivalently treated as either esophageal cancer or gastric cancer; independent descriptive statistics are consequently unavailable, and their actual status is poorly understood. The inclusion of this specific site of cancer in the standardized coding system will distinguish esophagogastric junction tumors.

## 4. Risk Factors of Gastric Cancer

The most reliable evidence base currently available, maintained by the IARC, classifies *H. pylori* infection and tobacco smoking as established causes of gastric cancer, with sufficient evidence for categorization as group 1 carcinogens [6,7]. Apart from these two factors, the most recently updated evaluation of risk factors for gastric cancer—summarized as an expert report in 2018 by the Continuous Update Project (CUP) of the World Cancer Research Fund (WCRF)/American Institute for Cancer Research (AICR) [8]—judged that there was strong evidence that consuming approximately three or more alcoholic drinks per day (greater than 45 g ethanol/day) and foods preserved by salting increase the risk of gastric cancer and that being overweight or obese, as assessed by body mass index (BMI), increases the risk of gastric cardia cancer. The expert panel also judged that there was some evidence to suggest that consuming grilled or barbecued meat and fish increases the risk of gastric cancer; consuming processed meat increases the risk of gastric non-cardia cancer; consuming little or no fruit increases the risk of gastric cancer; and consuming citrus fruit decreases the risk of gastric cardia cancer. A number of other factors were also assessed, but the evidence is limited, and no conclusions were drawn.

Table 1 summarizes current known risk factors for gastric cancer.

### 4.1. H. pylori Infection

**Figure 9:**
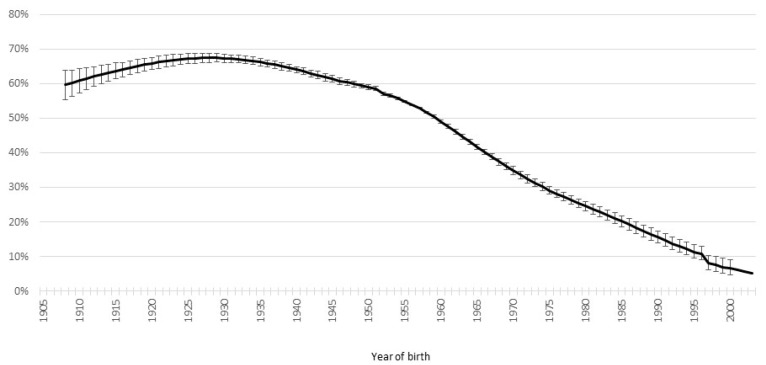
Declining trend in prevalence of *Helicobacter pylori* infection by birth year (1908–2003) in a Japanese population. Meta-analysis of 170,752 Japanese [10].

*H. pylori* remains the leading cause of gastric cancer, particularly non-cardia. Infection generally occurs in childhood, commonly before the age of 5, and is thus strongly associated with environmental hygiene during childhood, such as eating behaviors like mouth-to-mouth feeding [9]. These factors in infancy substantially determine the infection rate in adulthood. Of note, gastric cancer was the most common cancer in the last century in Japan, and the prevalence of infection with *H. pylori* has decreased with a birth cohort effect [10,11] with a peak at around 70–80% for people born in 1930–1940 but a decrease with age to only approximately 5% for people born around 2000 and without any substantial change in rate during life in each cohort (Figure 9). Moreover, estimates among Japanese children and adolescents clearly follow the trend in Japanese adults, in whom the prevalence of *H. pylori* was 10% for those born in 1985 but decreased to 3% for those born in 2011 [12]. This suggests that strategies to prevent gastric cancer have generational effects. Moreover, it also suggests that countries with similar experiences should investigate risk-stratified approaches to gastric cancer prevention in the coming decades. A similar decreasing trend has been seen in Korea [13,14]. National-level improvements in hygiene and overall socioeconomic status, generally influenced by a history of hygiene and health policy at the national level, will lead to a major decrease in the overall prevalence of *H. pylori* infection among all age groups, including countries with a high prevalence of infection. Infection will eventually become rare, and gastric cancer will commensurately decline.

The eradication of *H. pylori* infection is considered to be a preventive measure for gastric cancer, even considering possible adverse effects, including antibiotic resistance and gastroesophageal reflux diseases [15]. Importantly, the timing of eradication has a major effect on the efficacy of eradication in preventing gastric cancer [15]. Specifically, whether eradication is effective is considered dependent on its ability to reverse precancerous lesions (atrophic gastritis etc.) before the “point of no return”, or irreversible progression to gastric cancer [16]. A previous meta-analysis focusing on the “point of no return” reported that patients with intestinal metaplasia or dysplasia could not benefit from a decreased risk of gastric cancer by *H. pylori* eradication [17]. Accordingly, efficacy in preventing gastric cancer is considered to be attributable to the inhibitory effect of early eradication on the progression of these precancerous legions. To date, a series of systematic reviews and meta-analyses assessing the effectiveness of eradication in healthy asymptomatic individuals have shown a certain degree of evidence that eradication reduces the incidence of gastric cancer and death from gastric cancer, but these reports emphasize the need for additional research in different populations with different screening levels and longer follow-up periods beyond 10 years [18,19,20]. 

### 4.2. Tobacco Smoking

Tobacco smoking is an established cause of gastric cancer, with sufficient evidence for categorization by IARC as a group 1 carcinogen [6]. A systematic review and meta-analysis of prospective studies showed significantly increased risk for both sexes and for both cardia and non-cardia cancer in various populations. Current smokers had a 1.2- to 1.9-fold increased risk of gastric cancer compared with never-smokers in a dose-responsive manner [21]. Indeed, 16.5% of male and 1.9% of female gastric cancers were estimated to be attributable to tobacco smoking globally in 2020 [22].

The specific effect of tobacco smoking on gastric cancer is considered to be attributable to several mechanisms, in which nicotine, nitrosamines, and other nitroso compounds in smoke perturb gastric physiology [23]. Moreover, smoking slows gastric emptying and causes a consequent delay in alcohol absorption [24]. This also increases the risk of gastroesophageal reflux disease, which is associated with cardia cancer [25].

### 4.3. Alcohol Drinking

The IARC Monograph evaluation classifies alcoholic drinks and related acetaldehyde as group 1 carcinogens in humans [6]. According to evidence for intakes exceeding 45 g ethanol/day (approximately three drinks per day), increased alcohol consumption is a likely cause of gastric cancer, without a difference in the risk of cardia or non-cardia disease. Ethanol is fat-soluble, and its activity as a solvent increases the penetration of carcinogens into cells and damages the gastric mucosa [26]. Furthermore, the metabolite acetaldehyde has local toxic effects on the development of gastric cancer. The pathogenic effect of ethanol on gastric mucosal damage is related to the disruption of the gastric mucosal defense and external invasion and the balance between them. The incidence of alcohol dehydrogenase-2 (ALDH2) polymorphism and other genetic polymorphisms, which impede alcohol metabolism, is higher in East Asian populations [27].

### 4.4. Salt-Preserved Food

In general, high salt consumption is known to be related to hypertension and stroke as well as gastric cancer [28]. Whereas a high “amount” of salt consumption increases the risk of stroke, high consumption of foods preserved with salt increases the risk of gastric cancer rather than stroke [29]. The WCRF CUP Panel judged that a higher intake of salt-preserved foods is likely a cause of gastric cancer [8]. Highly salt-concentrated preserved foods irritate the lining of the stomach, facilitating or exacerbating *H. pylori* infection and leading to gastric cancer [30].

Salt reduction represents both a decrease in salt intake and a decrease in the intake of preserved foods having high salt concentrations. The consumption of salt-preserved foods was higher before the advent of refrigeration [31,32]. The spread of refrigeration over the last century substantially impacted the frequency of gastric cancer in some developed countries. Furthermore, the intake of fresh food has increased thanks to the growth of industrial refrigeration in both storage and transportation [31,32], which in turn reduced demand for salting and pickling and hastened the shift from salt preservation to frozen storage. This “unplanned triumph” of refrigeration was complemented by the adoption of population-based salt reduction strategies by both manufacturers and communities, demonstrating their efficacy in terms of both health and financial costs.

### 4.5. Obesity

Until recently, increased body fatness, as indicated by BMI, was not considered a risk factor for gastric cancer. One meta-analysis reported an increased risk of cardia cancer of 23% per 5 kg/m^2^ increase in BMI, although a meaningful association with non-cardia cancer was not seen. This impact of increased body fatness was particularly seen in men and in non-Asian or Western populations.

One characteristic of obesity is a low-grade chronic inflammatory state. The elevated production of pro-inflammatory factors like tumor necrosis factor (TNF)-alpha, interleukin (IL)-6 and C-reactive protein in chronic inflammation promotes cancer development. Obesity also results in increased levels of insulin and leptin and upregulates levels of endogenous hormones, including insulin and sex steroids. This may promote cell proliferation and decrease apoptosis, thereby promoting cancer cell growth. Moreover, obesity is associated with gastroesophageal reflux due to an increase in intraabdominal pressure, which is turn associated with the transition to possibly precancerous Barrett’s esophagus. By these mechanisms, obesity increases the risk of both cardia cancer and esophageal adenocarcinoma [8].

## 5. Future Trends

**Figure 10:**
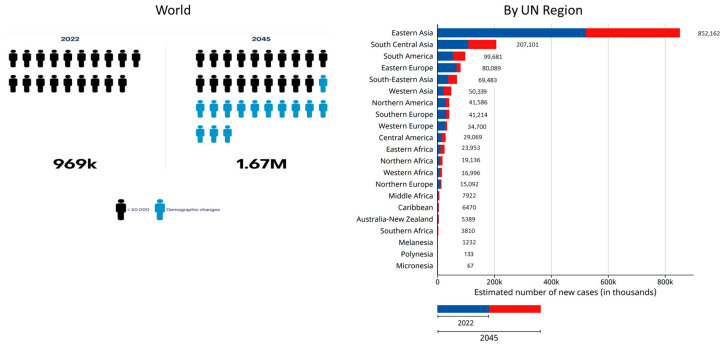
Estimated number of new cases of gastric cancer from 2022 to 2045 in both sexes by UN Region [33].

**Figure 11:**
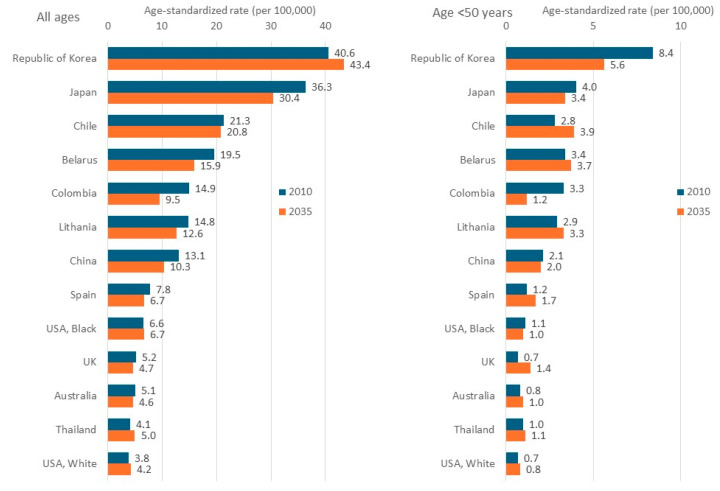
Age-standardized gastric cancer incidence rates (ASR) in 2010 and predicted rates in 2035 in both sexes combined [34].

The future burden of gastric cancer is of intense interest, as it is based on substantial changes in risk factors and environment over an extended period. The estimated incidence of gastric cancer from 2022 to 2045 indicates an increase in the number of new cases from 969,000 to 1.67 million worldwide and in most geographical regions (Figure 10) [33]. Focusing on the incidence rate, by contrast, provides a somewhat different perspective. A global assessment of the predicted incidence trend of gastric cancer from 2010 to 2035 showed that overall age-standardized incidence rates will continue falling in most countries, both high- and low-incidence. In many populations, by contrast, an increase in incidence rate is seen in younger groups (below 50 years) (Figure 11) [34]. This prediction implies that, in spite of the declining trend, more gastric cancer cases will occur in the next few decades due to aging and the growth of high-risk populations and that recent increases in younger ages among high-income countries indicate a generational transition in risk factors, from *H. pylori* and tobacco smoking, for example, to obesity [34].

## 6. Conclusions

Gastric cancer has generally shown a substantial decrease over the last century following decreases in risk factors, particularly *H. pylori* infection, tobacco smoking, and the intake of salt-preserved food. *H. pylori* eradication appears worthwhile as a preventive strategy in infected subjects at high risk, but longer follow-up is required. The continuing increase in obesity may result in increases in cardia cancer, particularly among Western populations, and should be carefully observed in both Western and Asian populations. These changes in gastric cancer epidemiology foreshadow a new era for gastric cancer control and warrant further monitoring of descriptive patterns and risk factors.

## Figures and Tables

**Table 1:** Summary of risk factors for gastric cancer [6,7,8].

Evidence Level		Risk Factor (Positive Association)
Strong Evidence	Convincing/ established cause	*Helicobacter pylori* infection (non-cardia) Tobacco smoking
	Probable	Body fatness (cardia) Alcoholic drinks (intake above 3 drinks or 45 g of ethanol per day) Food preserved by salting
Limited Evidence	Limited-suggestive	Processed meat (non-cardia) Grilled (broiled) or barbecued (charbroiled) meat and fish Low fruit intake Low citrus fruit (cardia)

## Data Availability

The original data presented in this review are available in references or cited URL.
